# Supplementary material for: Effects of Mars Global Simulant (MGS-1) on Growth and Physiology of Sweet Potato: A Space Model Plant
Source: Plants (Basel). 2023 Dec 23;13(1):55. doi: 10.3390/plants13010055 (PMC10780443; doi:10.3390/plants13010055)
Supplement: Supplementary file 1 [file plants-13-00055-s001.zip › plants-2746839-supplementary.pdf]

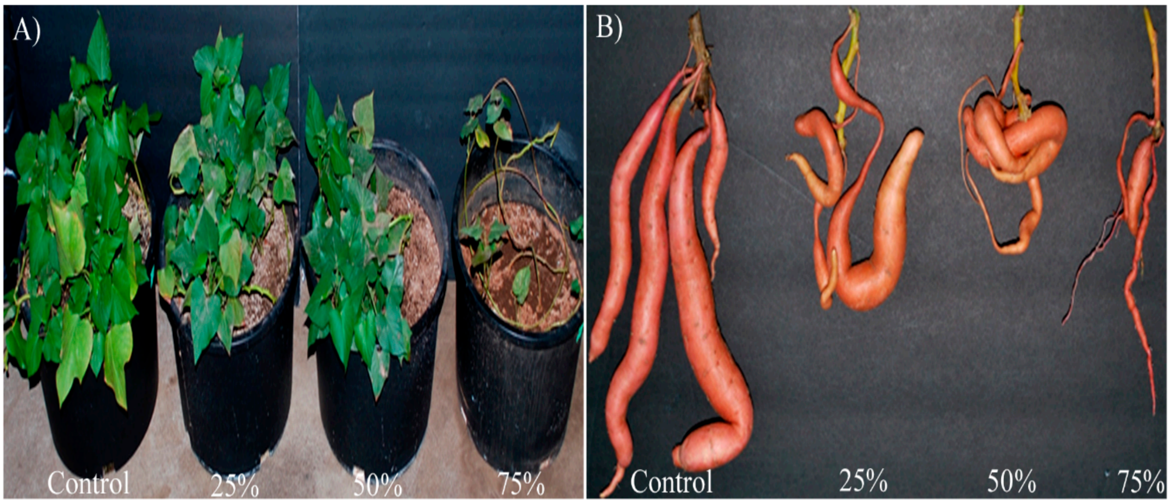

Figure S1: Effect of different concentrations of MGS1 exposure on A) growth and B) storage root and C) storage root fresh weight of *I. batatas*. Results are expressed as mean of triplicates ± SD. ***P* < 0.01, and ****P* < 0.001.

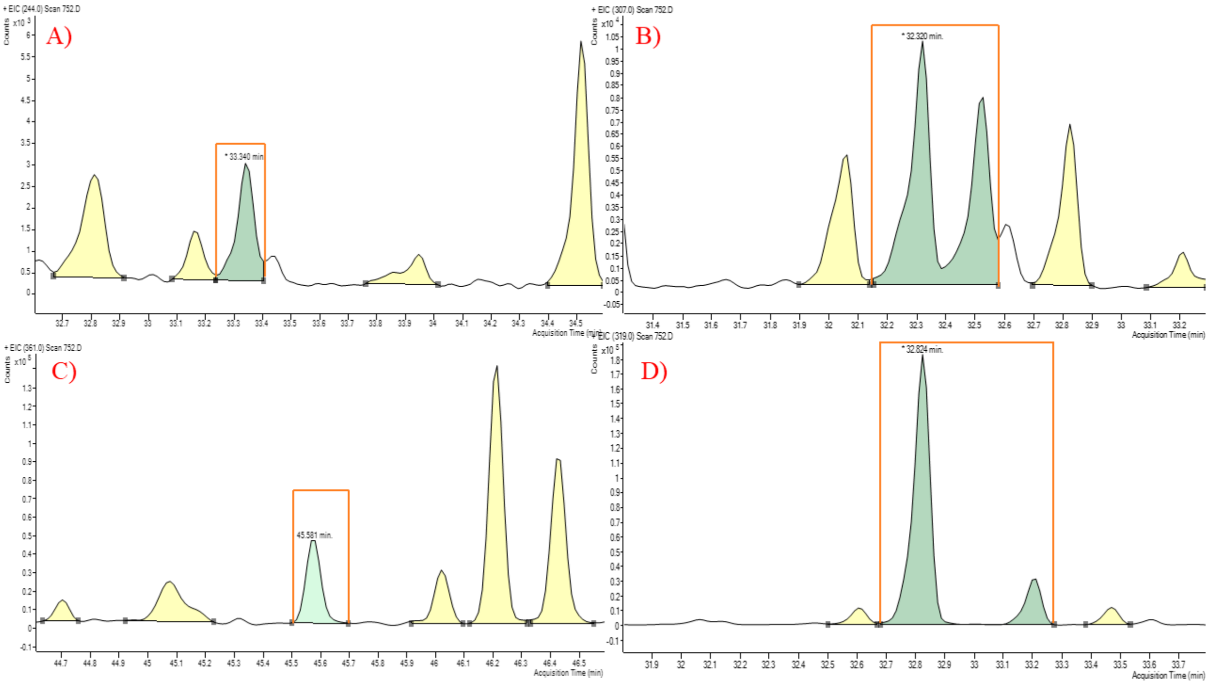

Figure S2: GC-MS chromatogram pattern of A) ascorbic acid, B) fructose, C) sucrose, and D) glucose.

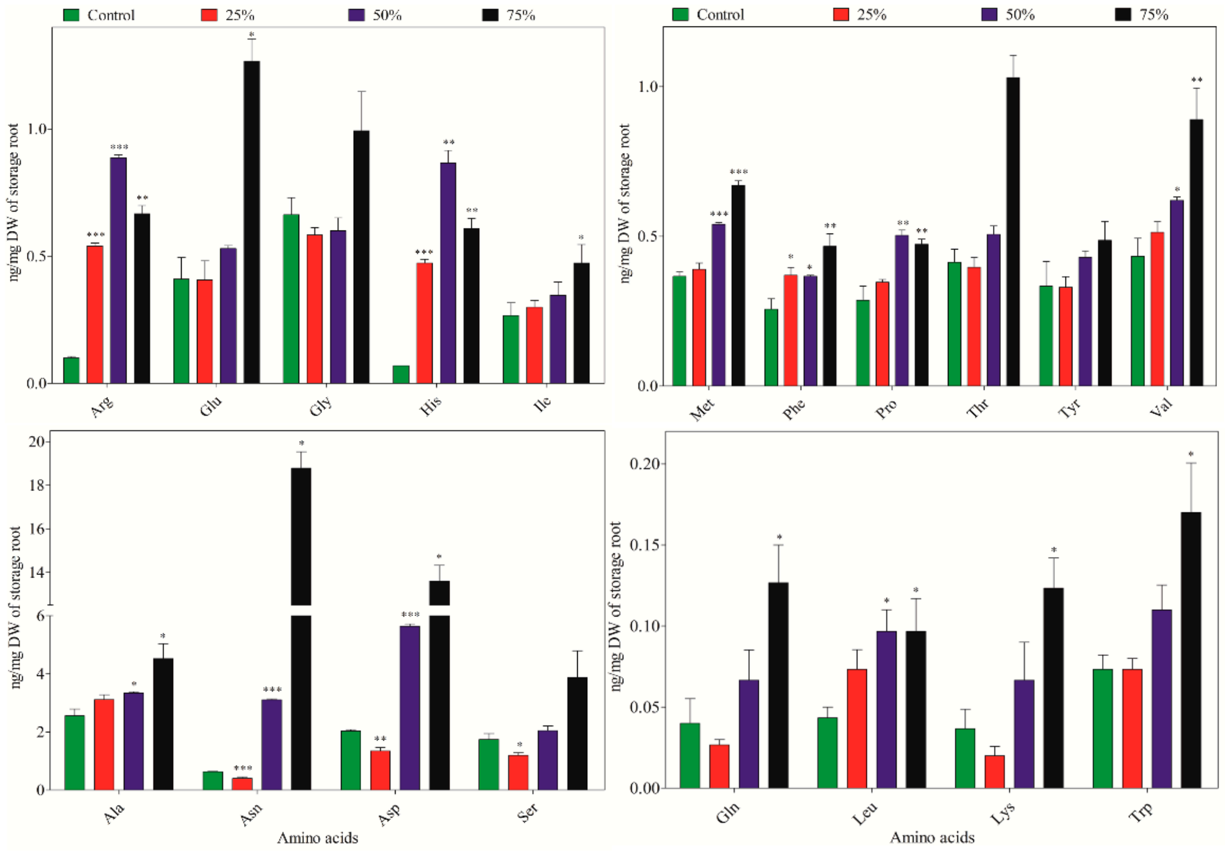

Figure S3: Effect of different MGS – 1 on amino acid profiling. Results are expressed as the means of three replicates ± SD and the asterisks indicate the significance at $*P < 0.05$, $**P < 0.01$, and $***P < 0.001$ compared to the control according to the independent samples t test.

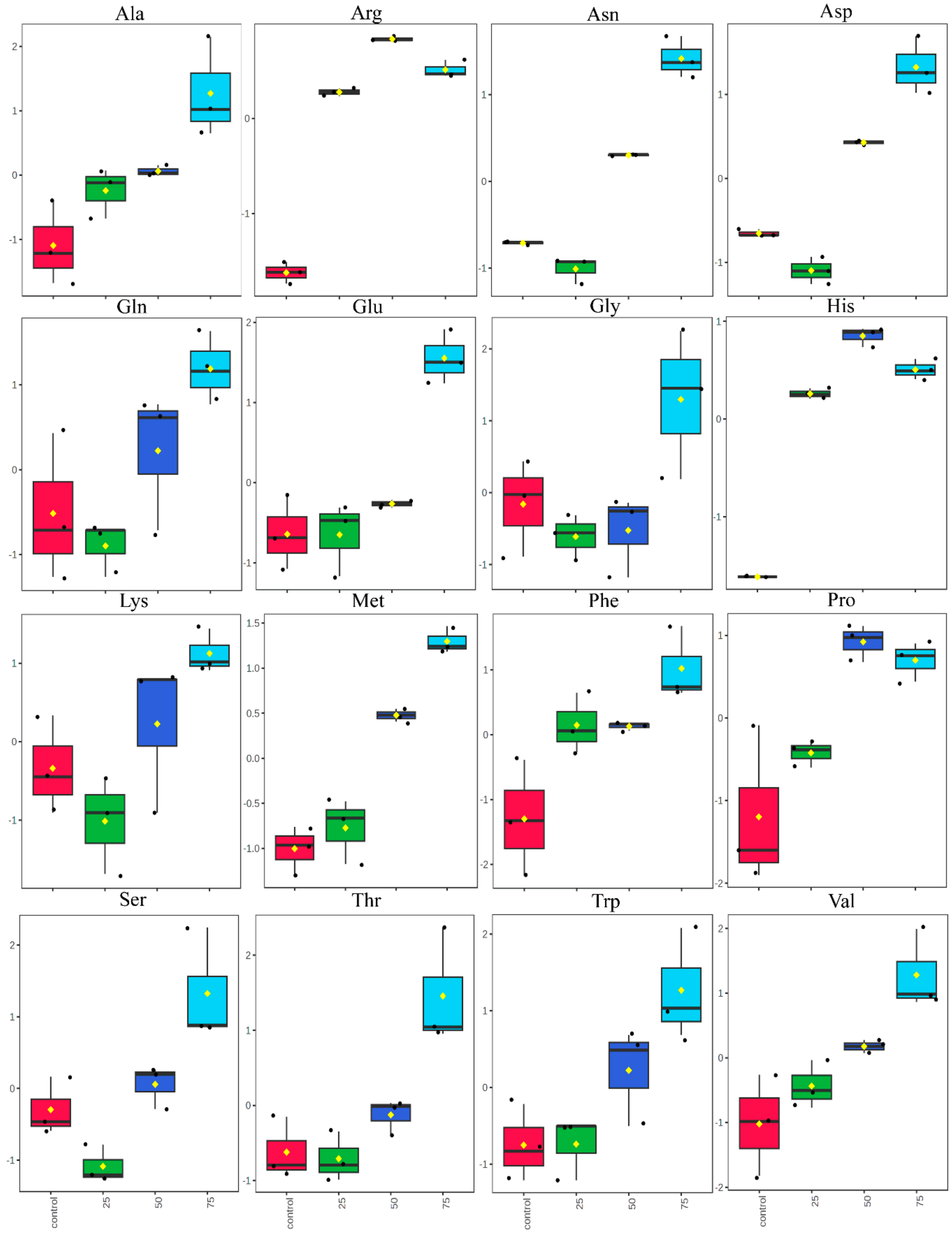

Figure S4: Box-Whisker plot for the significantly different amino acids for the different exposure of MGS – 1. The significantly different amino acids obtained from ANOVA and post-hoc analysis.

Sample preparation

Homogenized samples were spiked with internal standards (DL-Chlorophenyl alanine, and 13C Glc; both 0.01mg/mL), extracted with distilled water, centrifuged and separated onto two equal parts. One part was used for the amino acid quantitation, another – for sugar analysis.

Amino acids were analyzed by LCMS method

Chromatography was performed on Vanquish system (Thermo Scientific), with Hypersil Gold 2.1 x 150 mm (1.9µ) column; flow rate 600 µL/min. Mobile phases: 0.1% formic acid in water (A), 0.1% formic acid in acetonitrile (B). Gradient: 0-0.5min – 0%B, 0.5-3.5min – 60%B, 3.5-5.5min – 100%B, 5.5-7.5min – 0%B. Injection volume 1 µL. The column chamber temperature – 50^0C. Mass Spectrometry: TSQ Altis LC-MS/MS system (Thermo Scientific). Data acquired in positive SRM mode at 3500V (Table 1). Peak integration and quantitation done using Thermo TraceFinder (4.1) software.

Compound	Q1	Q3	Collision Energy (V)	RF Lens (V)
Gly	76	30	10.2	30.0
L-Ala	90	44	10.2	30.6
L-Arg	175	70	21.6	39.3
L-Asn	133	116	10.2	30.4
L-Asp	134	74	13.8	30.4
L-Cit	176	159	10.5	30.0
L-Gln	147	130	10.2	30.2
L-Glu	148	84	15.2	30.9
L-Ile	132	69	16.4	30.2
L-Leu	132	86	10.2	32.0
L-Lys	147	84	15.6	30.0
L-Met	150	104	10.2	30.0
L-Phe	166	103	26.9	30.6
L-Pro	116	70	14.3	31.1
L-Ser	106	60	10.2	30.6
L-Thr	120	102	10.2	30.4
L-Trp	205	188	10.2	31.1
L-Tyr	182	165	10.2	33.0
L-Val	118	55	19.6	30.0

Salvador AF, Askow AT, McKenna CF, Fang HY, Burke SK, Li Z, Ulanov AV, Paluska SA, Petruzzello SJ, Boppart MD, Oliver JM, Burd NA. Resistance Exercise-induced Regulation of Muscle Protein Synthesis to Intraset Rest. Med Sci Sports Exerc. 2020 May;52(5):1022-1030. doi: 10.1249/MSS.0000000000002213. PMID: 31703023.

GC/MS analysis

For sugar analysis supernatants were collected by centrifugation (5 min at 15,000 g), dried and derivatized with 75 µL methoxyamine hydrochloride (Sigma-Aldrich, MO, USA) (40 mg/ml in pyridine) for 60 min at 50 °C, then with 75 µL MSTFA+1%TMCS (Thermo, MA, USA) at 70 °C for 120 min, and following 2-hour incubation at room temperature. 10 µL of the internal standard (hentriacontanoic acid, 1 mg/mL) was added to each sample prior to derivatization. Chromatograms were acquired using a GC-MS system (Agilent Inc, CA, USA) consisting of an Agilent 7890 gas chromatograph, an Agilent 5975 MSD and a HP 7683B autosampler. Gas chromatography was performed on a ZB-5MS (60m×0.32mm I.D. and 0.25µm film thickness) capillary column (Phenomenex, CA, USA). The inlet and MS interface temperatures were 250°C, and the ion source temperature was adjusted to 230°C. An aliquot of 1 µL was injected with the split ratio of 10:1. The helium carrier gas was kept at a constant flow rate of 2 ml/min. The temperature program was: 5-min isothermal heating at 70°C, followed by an oven temperature increase of 5°C min^{-1} to 310°C and a final 10 min at 310°C. The mass spectrometer was operated in positive electron impact mode (EI) at 69.9 eV ionization energy at m/z 30-800 scan range combined with SIM mode. For SIM mode, following m/z fragments tracked: 307 (Fru), 319 (Glc), 361 (Suc), 244 (Ascorbic acid), 323 (13C6 Glc). Target peaks evaluated by the Mass Hunter Quantitative Analysis B.08.00 (Agilent Inc., CA, USA) software. Calibration curves generated with authentic standards for the 0.5– 50 µg/mL range.

To allow comparison between samples, all data were normalized to the internal standard in each chromatogram and the sample weight. The instrument variability was within the standard acceptance limit (5%).

ICP-MS analysis

1.0 SCOPE AND APPLICATION

1.1 Inductively coupled plasma-atomic emission spectrometry (ICP-AES) determines trace elements, including metals, in solution. The method is applicable to all of the elements listed in Table 1.

All matrices, excluding filtered groundwater samples but including ground water, aqueous samples, TCLP and EP extracts, industrial and organic wastes, soils, sludges, sediments, and other solid wastes require digestion prior to analysis.

Table 1 lists the elements for which this method is applicable, the recommended analytical wavelengths and estimated instrumental detection limits for the elements in clean aqueous matrices and method detection limit.

2.0 SUMMARY OF METHOD

2.1 Prior to analysis, samples must be solubilized or digested using appropriate Sample Preparation Method 3050B. When analyzing groundwater samples for dissolved constituents, acid digestion is not necessary if the samples are filtered and acid preserved prior to analysis.

2.2 This method describes multi-elemental determinations by ICP-AES using simultaneous optical systems and axial of the plasma. The instrument measures characteristic emission spectra by optical spectrometry. Samples are nebulized and the resulting aerosol is transported to the plasma torch. Element-specific emission spectra are produced by a radio-frequency inductively coupled plasma. The spectra are dispersed by a grating spectrometer, and the intensities of the emission lines are monitored by photosensitive devices. Background correction is required for trace element determination. Background must be measured adjacent to analyte lines on samples during analysis. The position selected for the background-intensity measurement, both sides of the analytical line, will be determined by the complexity of the spectrum adjacent to the analyte line. The possibility of additional interferences named in Section 3.0 should also be recognized and appropriate corrections made; tests for their presence are described in Section 7.5.

Safety Precautions

1. Safety glasses or goggles are mandatory when working in the laboratory. CONTACT LENSES ARE PROHIBITED!!
2. Lab coat and nitrile gloves mandatory.

3.0 INTERFERENCES

3.1 Spectral interferences are caused by background emission from the line emission of high concentration elements, overlap of a spectral line from another element, or unresolved overlap of molecular band spectra.

3.1.1 Background emission and stray light can usually be compensated for by subtracting the background emission determined by measurements adjacent to the analyte wavelength peak. Spectral scans of samples regions may indicate when alternate wavelengths are desirable because of severe spectral interference.

These scans will also show whether the most appropriate estimate of the background emission is provided by an interpolation from measurements on both sides of the wavelength peak or by measured emission on only one side. The locations selected for the measurement of background intensity will be determined by the complexity of the spectrum adjacent to the wavelength peak. The locations used for routine measurement must be free of off-line spectral interference (interelement or molecular) or adequately corrected to reflect the same change in background intensity as occurs at the wavelength peak. For multivariate methods using whole spectral regions, background scans should be included in the correction algorithm. Off-line spectral interferences are handled by including spectra on interfering species in the algorithm.

3.1.2 Spectral overlaps may be avoided by using an alternate wavelength.

4.0 APPARATUS AND MATERIALS

4.1 Inductively coupled argon plasma emission spectrometer (ICP-AES) SPECTRO CIROS (Spectro, Analytical Instruments).

4.1.1 Computer-controlled emission spectrometer with background correction.

4.1.2 Radio-frequency generator compliant with FCC regulations.

4.1.3 Autosampler AS-400 (Spectro, Analytical Instruments)

4.1.4 Argon gas supply--high purity Industrial Grade Liquid Argon, 180 Liter Cylinder (230 PSI) With Wheel Kit (Airgas East, Inc)

4.2 Volumetric flasks 500ml ±0.15, PYREXPLUS, class A

4.3 Volumetric pipettes 5mL, 0.5mL, 0.1mL, 10mL, KIMAX

5.0 REAGENTS

5. 1 DDI water

5.2 Standard IV certify solution by Inorganic Solution, Inc.

****The concentration of the standards solutions are located in Table 2.****

5.4 Standard IV Dilute to 500mL with 2 % Nitric acid. Mix calibration standard solution (IV) (Table 2). Fresh mixed standards should be prepared, as needed (indicate storage time).

5.4.1. Prepare Calibration Standards Solutions II, I diluted standard IV two times, four times and five times (Table 2).

5.5 Two types of blanks are required for the analysis for samples prepared. The calibration blank is used in establishing the analytical curve, and the method blank is used to identify possible contamination resulting from varying amounts of the acids used in the sample processing.

5.5.1 The calibration blank is prepared by acidifying DDI water to the same concentrations of the acids found in the standards and samples.

5.5.2 The method blank must contain all of the reagents in the same volumes as used in the processing of the samples. The method blank must be carried through the complete procedure and contain the same acid concentration in the final solution as the sample solution used for analysis.

6.0 PROCEDURE

6.1 Preliminary treatment of most matrices is necessary because of the complexity and variability of sample matrices. Groundwater samples which have been profiltered and acidified will not need acid digestion. Samples which are not digested must be matrix matched with the standards**.** Solubilization and digestion procedures are presented in Sample Preparation Methods.

6.2 Set up the instrument with proper operating parameters established as detailed below. The instrument must be allowed to become thermally stable before beginning, at least 60 minutes of operation prior to calibration.

Equipment Operation:

Forward power - 1.4kw

Coolant gas - 13.5LPM

Plasma gas - 1.2LPM

Sample gas - 0.8LPM

Sample uptake rate - 2.2mL/min using a peristaltic pump and a fixed cross flow nebulizer

6.2.1 After completing the initial optimization of operating conditions begin daily calibration. The load method is "Soil 2021 " and the instrument is profiled using a multielement standard IV

6.2.2 This is necessary to align the entrance slit so that all of spectral lines are centered on their respective parabola-picks there by yielding maximum and corrected background points.

6.2.3 The instrument is calibrated with 4 multielement standard solution and one reagent blank 10% Nitric acid on Table2

6.2.4 Before using this procedure to analyze samples, there must be data available documenting initial demonstration of performance. These documented data are in the file. The filename has the same name (Field number) which samples are received when are submitted.

6.3 Rinse the system with the calibration blank solution before the analysis of each sample. The rinse time will be one minute.

6.4 The sample concentration is calculated by instrument software and reported: concentration (mg/L or mg/kg).

7.0 QUALITY CONTROL

7.1 All quality control data should be maintained and available for easy reference

7.2 Dilute and reanalyze samples that exceed the linear calibration range or use an alternate, less sensitive line for which quality control data is already established.

7.3 Employ a minimum of one method blank per sample batch to determine if contamination or any memory effects are occurring. A method blank is a volume of reagent water carried through the same preparation process as a sample

7.4 Analyze matrix spiked duplicate samples at a frequency of one per matrix batch. matrix duplicate sample is a sample brought through the entire sample preparation and analytical process in duplicate

7.4.1. The spiked sample or spiked duplicate sample recovery is to be within ± 25% of the actual value or within the documented historical acceptance limits for each matrix (Table 4).

7.5 It is recommended that whenever a new or unusual sample matrix is encountered, a series of tests be performed prior to reporting concentration data for analyte elements. These tests, as outlined in Sections 7.5.1 and 7.5.2, will ensure that neither positive nor negative interferences are operating on any of the analyte elements to distort the accuracy of the reported values.

7.5.1 Dilution Test: If the analyte concentration is sufficiently high (minimally, a factor of 10 above the instrumental detection limit after dilution), an analysis of a 1:5 dilution should agree within ±10% of the original determination.

7.5.2 Post Digestion Spike Addition: An analyte spike added to a portion of a prepared sample, or its dilution should be recovered to within 75% to 125% of the known value. The spike addition should produce a minimum level of 10 times and a maximum of 100 times the instrumental detection limit. If the spike is not recovered within the specified limits, a matrix effect should be suspected (Table 3).

CAUTION: If spectral overlap is suspected, use of computerized compensation, an alternate wavelength, or comparison with an alternate method is recommended

7.6 Check the instrument standardization by analyzing appropriate QC samples as follows.

7.6.1 Verify calibration with the Continuing Calibration Verification (CCV) Standard immediately following daily calibration, after every ten samples, and at the end of an analytical run. Check calibration with the Initial Calibration Verification (ICV) following the initial calibration At the laboratory's discretion, an ICV may be used in lieu of the continuing calibration verifications (CCV). If used in this manner, the ICV should be at a concentration near the mid-point of the calibration curve. Use a calibration blank immediately following daily calibration, after every 10 samples and at the end of the analytical run.

7.6.1.1 The results of the ICV and CCVs are to agree within 10% of the expected value; if not, terminate the analysis, correct the problem, and recalibrate the instrument. Table 5 (Precision and Accuracy Data).

7.6.1.2 The results of the check standard are to agree within 10% of the expected value; if not, terminate the analysis, correct, and recalibrate the instrument.

7.6.1.3 The results of the calibration blank are to agree within three times the IDL. If not, repeat the analysis two more times and average the results. If the average is not within three standard deviations of the background mean, terminate the analysis, correct the problem, recalibrate, and reanalyze the previous 10 samples. If the blank is less than 1/10 the concentration of the action level of interest, and no sample is within ten percent of the action limit, analyses need

not be rerun and recalibration need not be performed before continuation of the run. Table 6 the result of blank sample.

7.6.2 Verify the interelement and background correction factors at the beginning of each analytical run. Do this by analyzing the interference check sample. Results should be within ± 20% of the true value.

Note: A waste tube of ICP-AES must be into label container with calcium carbonate.

8. REFERENCES

1. Boumans, P.W.J.M. Line Coincidence Tables for Inductively Coupled Plasma Atomic Emission Spectrometry, 2nd Edition. Pergamon Press, Oxford, United Kingdom, 1984.

2. Sampling and Analysis Methods for Hazardous Waste Combustion; U.S. Environmental Protection Agency; Air and Energy Engineering Research Laboratory, Office of Research and Development: Research Triangle Park, NC, 1984; Prepared by Arthur D. Little, Inc.

3. Rohrbough, W.G.; et al. Reagent Chemicals, American Chemical Society Specifications, 7th ed.; American Chemical Society: Washington, DC, 1986.

4. 1985 Annual Book of ASTM Standards, Vol. 11.01; "Standard Specification for Reagent Water"; ASTM: Philadelphia, PA, 1985; D1193-77.

5. Jones, C.L. et al. An Interlaboratory Study of Inductively Coupled Plasma Atomic Emission Spectroscopy Method 6010 and Digestion Method 3050. EPA-600/4-87-032, U.S. Environmental Protection Agency, Las Vegas, Nevada, 1987.

Table S1: Wavelengths and Method Detection Limit (MDL).

Detection Element	Wavelengths (mm)	MDL
**Aluminum**	308.215	0.277
**Arsenic**	189.042	0.053
**Barium**	455.404	0.003
**Beryllium**	313.042	0.001
**Boron**	249.773	0.167
**Cadmium**	228.802	0.004
**Calcium**	211.276	0.225
**Calcium**	317.933	0.132
**Chromium**	267.716	0.010
**Cobalt**	228.615	0.008
**Copper**	324.754	0.005
**Iron**	275.573	0.125
**Lead**	220.351	0.035
**Lithium**	670.784	0.002
**Magnesium**	279.079	0.069
**Manganese**	293.93	0.006
**Molybdenum**	202.03	0.038
**Nickel**	231.604	0.028
**Phosphorus**	213.618	0.033
**Potassium**	766.49	0.040
**Sodium**	589.592	0.023
**Strontium**	421.552	0.003
**Sulfur**	182.034	0.107
**Titanium**	334.941	0.030
**Vanadium**	292.402	0.036
**Zinc**	213.856	0.032

Table S2: Standard solution preparation for CNAL Procedure S2020: Microwave HNO_3 digestion for soil samples.

**10% HNO_3**				
	for 500mL	**Std IV**	**Std II**	**Std I**
**Elements**	**mL stock**	**Conc.**	**Conc.**	**Conc.**
**Al**	25	500	100	50
**As**	0.1	2	0.4	0.2
**B**	0.5	5	1	0.5
**Ba**	5	10	2	1
**Be**	1	2	0.4	0.2
**Ca**	25	500	100	50
**Cd**	0.1	2	0.4	0.2
**Co**	0.1	2	0.4	0.2
**Cr**	1	20	2	2
**Cu**	0.5	10	2	1
**Fe**	25	500	100	50
**K**	10	200	40	20
**Li**	0.25	5	1	0.5
**Mg**	10	200	40	20
**Mn**	1	20	4	2
**Mo**	0.1	2	0.4	0.2
**Na**	2.5	50	10	5
**Ni**	0.25	5	1	0.5
**P**	5	100	20	10
**Pb**	0.5	10	2	1
**S**	2.5	50	10	5
**Sb**	1	2	0.4	0.2
**Se**	1	2	0.4	0.2
**Sr**	0.5	10	2	1
**Ti**	5	100	20	10
**V**	0.2	2	0.4	0.2
**Zn**	2.5	50	10	5

Table S3: Spiked sample preparation using CNAL Procedure S2020 using Spectro CIROS CCD ICP.

**2% HNO_3**			
	**100mL stock**	**con 100mL**	**final con**
**Elements**			mg/L
**Al**	10	1000	100
**As**	0.2	20	2
**B**	0.1	30	3
**Ba**	5	50	5
**Be**	2	20	2
**Ca**	10	1000	100
**Cd**	0.2	20	2
**Co**	0.2	20	2
**Cr**	2	200	20
**Cu**	1	100	10
**Fe**	10	1000	100
**K**	10	1000	100
**Li**	0.5	50	5
**Mg**	10	1000	100
**Mn**	2	200	20
**Mo**	0.2	20	2
**Na**	2	200	20
**Ni**	0.5	50	5
**P**	5	500	50
**Pb**	1	100	10
**S**	5	500	50
**Sr**	1	100	10
**Ti**	5	500	50
**V**	0.4	20	2
**Zn**	5	500	50

Table S4: Quality assurance objective soil spike samples by measurement using CNAL Procedure S2020 and the Spectro CIROS CCD ICP.

	Master Soil Sample: AuroraIII				Master Soil Sample: Mahaska			
**Element (wavelength)**	**Recovery (%)**	**RSD (%)**	**Acceptance range**		**Recovery (%)**	**RSD (%)**	**Acceptance range**	
**Al308.215**	103.49	8.79	125	81	115.46	2.40	124	107
**As189.042**	99.27	0.02	91	90	1.10	2.91	1	1
**B249.773**	105.79	0.21	105	104	108.57	0.15	109	108
**Ba455.404**	92.95	0.05	93	93	83.77	0.38	85	83
**Be313.042**	97.79	0.10	98	98	109.48	0.06	110	109
**Ca211.276**	98.62	7.83	117	79	123.36	0.31	125	122
**Ca317.933**	98.62	7.29	115	80	122.11	0.36	123	121
**Cd228.802**	101.11	0.08	102	102	115.46	0.07	116	115
**Co228.615**	94.89	0.08	95	95	104.80	0.05	105	105
**Cr267.716**	97.60	0.87	100	96	108.77	0.63	111	107
**Cu324.754**	96.26	0.39	98	96	108.58	0.31	110	108
**Fe275.573**	91.28	11.57	114	63	121.19	0.70	124	119
**K766.490**	101.24	7.05	118	82	122.58	0.08	123	122
**Li670.784**	95.96	0.03	80	77	95.24	0.26	96	95
**Mg279.079**	104.73	6.89	123	87	119.28	0.78	122	116
**Mn293.930**	88.48	0.40	92	90	91.87	0.25	93	91
**Mo202.030**	95.94	0.09	96	96	110.57	0.07	111	110
**Na589.592**	98.45	2.59	100	87	120.27	0.11	121	120
**Ni231.604**	94.92	0.24	96	94	108.01	0.19	109	107
**P213.618**	90.25	2.44	93	78	121.79	2.64	131	112
**Pb220.351**	99.67	0.22	78	68	101.15	0.42	102	100
**S182.034**	94.40	2.07	100	90	104.18	1.23	108	100
**Sr421.552**	95.92	0.46	97	95	109.89	0.36	111	109
**Ti334.941**	82.68	2.34	86	73	119.75	2.27	128	112
**V292.402**	95.05	0.06	96	96	101.34	0.02	101	101
**Zn213.856**	98.03	2.55	273	124	115.48	0.41	117	114

Table S5: Spectro CIROS CCD ICP precision and accuracy data.

	**Master Soil Sample: Mahaska**		**Master Soil Sample: Aurora-III**	
	**Average (mg/kg)**	**RSD (%)**	**Average (mg/kg)**	**RSD (%)**
**Al308.215**	11377.31	8.81	8280.78	8.70
**As189.042**	3.49	7.25		
**B249.773**	10.37	9.44	12.82	7.27
**Ba455.404**	210.70	4.02	38.88	8.50
**Be313.042**	0.63	4.38	0.44	8.39
**Ca211.276**	4360.51	3.89	1642.33	8.17
**Ca317.933**	4461.46	4.14	1652.15	8.26
**Co228.615**	5.03	9.11	7.32	7.73
**Cr267.716**	12.92	9.58	11.16	8.60
**Cu324.754**	13.29	6.53	9.68	5.88
**Fe275.573**	13893.98	6.80	15307.39	6.07
**K766.490**	1516.03	8.31	539.04	4.78
**Li670.784**	6.23	10.76	11.45	6.16
**Mg279.079**	2270.88	5.05	2446.73	9.16
**Mn293.930**	472.59	7.48	324.76	7.38
**Na589.592**	36.78	12.91	18.69	8.20
**Ni231.604**	10.13	10.00	14.28	9.99
**P213.618**	477.65	4.94	376.76	8.20
**Pb220.351**	10.30	7.08	7.54	6.66
**S182.034**	233.85	4.01	137.16	7.91
**Sr421.552**	18.13	5.51	4.14	5.74
**Ti334.941**	25.32	9.84		
**V292.402**	20.63	9.32	12.52	8.46
**Zn213.856**	52.62	5.32	46.86	5.30

Table S6: Blank sample results using CNAL Procedure S2020.

Element	IDL	3*LDL	Blank
	µg/mL	µg/mL	µg/mL
**Al 308.215**	0.013	0.039	0.000
**As 89.042**	0.012	0.035	0.000
**Ba455.404**	0.003	0.008	0.000
**Be313.042**	0.000	0.001	0.000
**B 249.773**	0.001	0.004	0.000
**Cd226.502**	0.000	0.001	0.000
**Ca 11.276**	0.031	0.092	0.000
**Ca 17.933**	0.012	0.036	0.000
**Cr 67.716**	0.007	0.021	0.000
**Co 28.615**	0.001	0.002	0.000
**Cu 24.754**	0.001	0.002	0.000
**Fe 75.573**	0.011	0.032	0.000
**Pb220.351**	0.018	0.055	0.000
**Li670.784**	0.002	0.006	0.000
**Mg 79.079**	0.006	0.019	0.000
**Mn 93.930**	0.002	0.006	0.000
**Mo 02.030**	0.002	0.007	0.000
**Ni 231.604**	0.002	0.007	0.000
**P213.618**	0.008	0.024	0.000
**K 766.490**	0.002	0.006	0.000
**Na 89.592**	0.001	0.003	0.000
**Sr421.552**	0.000	0.001	0.000
**S182.034**	0.011	0.034	0.000
**Ti 334.941**	0.003	0.009	0.000
**V 292.402**	0.001	0.004	0.000
**Zn 13.856**	0.001	0.004	0.000

Master Soil Aurora V

Sample Name	Aurora V	RSD%
	mg/Kg	
Al308.215	11109.04	6.34
As189.042	3.21	20.32
B249.773	10.85	13.89
Ba455.404	76.08	6.96
Be313.042	0.59	7.42
Ca211.276	3271.72	7.36
Ca317.933	3253.27	7.91
Cd228.802	-	-
Co228.615	8.17	7.63
Cr267.716	16.05	9.13
Cu327.396	15.46	7.04
Fe275.573	19749.71	6.05
K766.490	1088.86	9.12
Li670.784	23.70	8.21
Mg279.079	3151.98	5.89
Mn257.610	728.20	5.72
Mo202.030	-	-
Na589.592	36.77	10.53
Ni231.604	19.62	8.10
P213.618	609.98	9.65
Pb220.351	13.78	9.94
S182.034	291.94	6.74
Sr421.552	7.96	7.22
Ti334.941	18.09	9.59
V292.402	15.36	7.33
Zn213.856	78.46	8.20
